# Working Health Services Scotland: a 4-year evaluation

**DOI:** 10.1093/occmed/kqx186

**Published:** 2018-01-30

**Authors:** E Demou, M Hanson, A Bakhshi, M Kennedy, E B Macdonald

**Affiliations:** 1 MRC/CSO Social and Public Health Sciences Unit, Institute of Health and Wellbeing, University of Glasgow, Glasgow, UK; 2 Healthy Working Lives Group, Public Health, Institute of Health and Wellbeing, University of Glasgow, Glasgow, UK; 3 WorksOut, The Green House, Edinburgh, UK; 4 Salus Occupational Health, Safety & Return to Work Services, Hamilton, UK

**Keywords:** Case management, intervention, mental health, musculoskeletal disorders, return-to-work, sickness absence, SME

## Abstract

**Background:**

Working Health Service Scotland (WHSS) supports the self-employed and employees of small and medium-sized enterprises (SMEs) in Scotland with a health condition affecting their ability to work, who are either absent or at risk of becoming absent due to it.

**Aims:**

To evaluate the impact on health and work outcomes of WHSS clients over a 4-year period.

**Methods:**

Data were collected at enrolment, entry, discharge and follow-up at 3 and 6 months after discharge. Clients completed up to three validated health questionnaires at entry and discharge—EuroQol five dimensions (EQ-5D) and visual analogue scale (VAS); Hospital Anxiety and Depression Scale (HADS); and Canadian Occupational Performance Measure (COPM).

**Results:**

A total of 13463 referrals occurred in the 4-year period; 11748 (87%) were eligible and completed entry assessment and 60% of the latter completed discharge paperwork. The majority of referrals were due to musculoskeletal conditions (84%) while 12% were referred with mental health conditions. Almost a fifth (18%) of cases were absent at entry and back at work at discharge. Work days lost while in WHSS was associated with age, length of absence prior to entering WHSS, primary health condition and time in programme. All health measures showed significant improvements from entry to discharge. Improvement in general health was sustained at 3- and 6-month follow-up.

**Conclusions:**

The WHSS evaluation findings indicate that participation was associated with positive changes to health and return-to-work. The extent of the positive change in health measures and work ability can be highly important economically for employees and employers.

## Introduction

Sickness absence (SA) is a public health issue with substantial impacts on employers, employees, health care systems and society [1–3]. The longer someone is absent, the higher the risk of not returning to work [3]. As SA is multicausal, the biopsychosocial (BPS) model has been used in the management of SA [4] and is recognized by the World Health Organization (WHO) [5] as an appropriate model.

Mental health (MH) and musculoskeletal disorders (MSD) are the leading cause of long-term SA [1,2]. These are of particular interest because improving return-to-work (RTW) times is one of the main goals of SA interventions. Although only responsible for a small percentage of SA events, long-term SA events account for ~75% of absence costs [2,6]. Workplace interventions targeted at improving physical and psychosocial health [7–9], quality of life [10], emotional well-being [11], presenteeism [11], absenteeism [12] and SA [13] are effective. The workplace is often a conducive environment to implement programmes and practices to promote employee health and well-being [14].

Employees of small and medium-sized enterprises (SMEs) and the self-employed are most likely to have little or no formal occupational health (OH) support [15]. A study on the health and well-being needs of SME employees found SA and presenteeism to be two emerging themes [14]. The characteristics, working patterns and culture in SMEs further impact on absenteeism and presenteeism rates [16]. The size of the SME, and their knowledge of OH and its importance, influence provision of OH services in SMEs [17]. A large number of SMEs understand and monitor SA and its impact on their organization [17], whereas only a small fraction undertake health promotion activities [15]. SME-specific information about the occurrence of SA and work attendance behaviour when dealing with a health issue and the variables that influence these decisions is needed for the development of tailored interventions for this sector [16].

The Working Health Service Scotland (WHSS) programme, funded by the Scottish Government and UK Department for Work and Pensions (DWP), was developed to help meet this need within SMEs [18]. It provides telephone-based case management and some face-to-face therapeutic support to SME employees whose health condition affects their ability to work [19]. WHSS aims to provide support for health conditions which may impact on the clients’ work and/or other areas of their lives, to allow them to RTW (if absent) or remain in work (if not absent).

The service is provided in 11 health board areas in Scotland (out of 14), with clients from outside these areas being managed by other boards [18]. The self-employed and SME (<250 employees) employees in Scotland can self-refer, or be referred by a general practitioner (GP) or allied health professional (AHP); employers cannot make referrals. WHSS uses a BPS approach with an occupational focus and provides physiotherapy, occupational therapy and psychological therapy/counselling where appropriate. Care is coordinated by a case manager.

This study aimed to investigate the health and work/functional outcomes of WHSS clients referred between 2010 and 2014 and understand the factors associated with programme completion and RTW.

## Methods

Client data enrolled between 26 March 2010 and 31 March 2014 (including discharge data to 28 July 2014) were analysed [18]. Data collection points included: *enrolment* (WHSS eligibility assessment, i.e. working for a Scottish SME); *entry* (first telephone assessment by case manager—‘pre-intervention’); *therapy provision* (services received); *discharge* (discharge from WHSS—‘post-intervention’); *3-month post-discharge follow-up* (recording health measures, work ability and absence status); and *6-month post-discharge follow-up* (repeat of data gathered at 3-month follow-up). Demographic, health (presenting conditions) and employment status (at work/absent) data were collected at entry. Clients also completed up to three standard, validated health questionnaires at entry and discharge—the EuroQol five dimensions questionnaire (EQ-5D) and visual analogue scale (EQ-5D VAS), the Hospital Anxiety and Depression Scale (HADS) and the Canadian Occupational Performance Measure (COPM). EQ-5D was included in the 3- and 6-month follow-up [8].

Clients completed telephone assessments to establish eligibility and provide consent, followed by a telephone assessment by the case manager [18]. Where required, clients were referred to services either within or external to WHSS (appointments within 5–10 working days from referral). Case managers monitored progress and clients were discharged when suitable improvements in health/work ability were achieved or if WHSS was no longer supporting them adequately. Discharged clients were followed up 3 and 6 months post-discharge.

Descriptive statistics analysed demographics, health condition, and health assessment scores pre-/post-intervention and at follow-up. Univariate analysis was used to investigate differences pre-/post-intervention. A general linear model was used to examine significant predictors of the degree of change in the health assessment scores. Multivariate statistical analysis was used to investigate the relationships between the routinely collected variables and explore which predictor variables were associated with the outcome variable(s). Predictor variables included age, gender, deprivation (Scottish Index of Multiple Deprivation, SIMD), health condition and work status. For clients who completed WHSS, number of therapeutic sessions attended, programme duration and therapies received were also predictor variables. The outcome variable was programme completion (dichotomous: completed/discharged); and for those who completed the difference in pre- and post-intervention health assessment scores was investigated. In a second analysis, the outcome was work status (dichotomous: in work/absent) at time of exit from WHSS.

This is a retrospective study using secondary anonymized data. Ethical approval was not required as confirmed by the NHS Lanarkshire Research & Development Manager.

## Results

A total of 13463 referrals were made over the study period, with 11748 cases (87%) eligible with completed entry assessments. Some individuals entered the programme more than once, so the number of unique individuals (i.e. ‘clients’) is less than the number of referrals (i.e. ‘cases’). The majority (92%) of referrals were new referrals; 5% were re-referring with a new health condition; and 3% were re-referring with the initial health condition. The analysis hereafter is based on the number of ‘cases’ and not ‘clients’, so each individual referral is considered unique. Overall 7% of females and 6% of males re-entered WHSS, with older clients more likely to re-enter [18].

The number of cases significantly increased with increasing SIMD category (*P <* 0.01) (Table 1). Most demographic data were relatively similar across SIMD quintiles, with the exception of age and absence status at entry (Table 1). The greatest proportion of cases (21%) came from the standard occupational classification (SOC) group SOC-5: skilled trades occupations, while the smallest (7%) came from SOC-8: process, plant and machine, and SOC-9: elemental occupations. The majority of cases were referred with a MSD (84%); while 12% were referred with a MH condition. All other health conditions were categorized as ‘Other’ (4%). This paper focuses on MSD and MH cases.

**Table 1.** Demographic details at entry by SIMD quintiles

Cases pre-intervention	SIMD quintiles					Whole sample
	1 (most deprived)	2	3	4	5 (least deprived)	
Number	1718	2228	2467	2626	2709	11748
Average age (SD) (years)	43.3 (12)	43.5 (12)	44.6 (12)	45.7 (12)	45.4 (12)	44.6 (12)
Gender						
Female (%)	49	47	49	48	48	48
Male (%)	50	52	50	51	51	51
Missing/not specified (%)	0.4	0.8	0.5	0.7	0.8	0.6
Primary health condition						
MSD (%)	85	83	83	85	85	84
MH (%)	12	14	13	11	11	12
Other (%)	3	3	4	4	4	4
Employment status						
Full-time (%)	74	77	75	78	75	76
Part-time (%)	26	23	25	22	25	24
Absent at entry (%)	24	22	22	21	19	21

There was a significant association between deprivation (SIMD) and primary condition (*P <* 0.05); cases in SIMD 2 and 3 were 1.2 and 1.1 times more likely, respectively, to have MH as their primary condition compared to the least deprived (SIMD 5). There was also a strong association between gender and primary condition (*P* < 0.01), with women twice as likely as men to present with a MH condition (relative risk [RR] = 2.00; 95% CI: 1.81 to 2.22). Type of occupation was also associated with primary condition (*P* < 0.01); all occupational groups had a higher risk of MH conditions compared to SOC-5, with the associate professional and technical occupations group (SOC-3) having the highest risk overall (three times higher risk of MH conditions compared to SOC-5).

A secondary health condition was reported by 2154 cases (16%); 24% of MH cases had a secondary health condition (most commonly another MH condition), while 15% of MSD cases had a secondary condition (most commonly another MSD) (Table S1, available as Supplementary data at *Occupational Medicine* Online).

A quarter of cases (*n* = 2902) were absent at entry. By primary health condition, 21% (*n* = 2121) of MSD cases and almost double (41%; *n* = 589) of MH cases were absent at entry. A total of 2145 cases provided information on absence duration prior to entry to WHSS, with 36% absent for <2 weeks and 20% absent for >12 weeks (Figure S1, available as Supplementary data at *Occupational Medicine* Online).

Forty-one per cent of cases who entered the programme did not complete the discharge paperwork. This analysis is based on the data received from those who completed at least some of the discharge paperwork. The average time in WHSS was 121 ± 81 days (Table S2, available as Supplementary data at *Occupational Medicine* Online). Overall, half (50%) of the 11103 cases, for whom there were entry EQ-5D scores, completed the discharge EQ-5D. Socio-demographic characteristics between those that completed the programme were similar to the entire WHSS population for gender, primary health condition, SIMD and absence status at entry. Multivariate analysis showed that age was a significant predictor of completion (*P* < 0.001) and number of services and longer duration in WHSS were inversely associated with the odds of completing discharge paperwork (*P* < 0.001) [18].

The majority of cases (75%) were at work at entry and discharge, while 4% were absent at entry and discharge. However, 18% (*n* = 1188) who were absent at entry were at work at discharge. Two per cent were at work at entry and absent at discharge. Cases more likely to be absent at discharge included cases: in the most deprived compared to least deprived group (RR = 1.89; 95% CI: 1.42 to 2.53); aged over 50 compared to 30–39 years old group (RR = 1.44; 95% CI: 1.09 to 1.92); with MH rather than MSD conditions (RR = 1.89; 95% CI: 1.50 to 2.39); and cases absent at entry were six times more likely to be absent at discharge compared to those at work at entry (RR = 6.04; 95% CI: 5.00 to 7.30).

**Figure 1.**
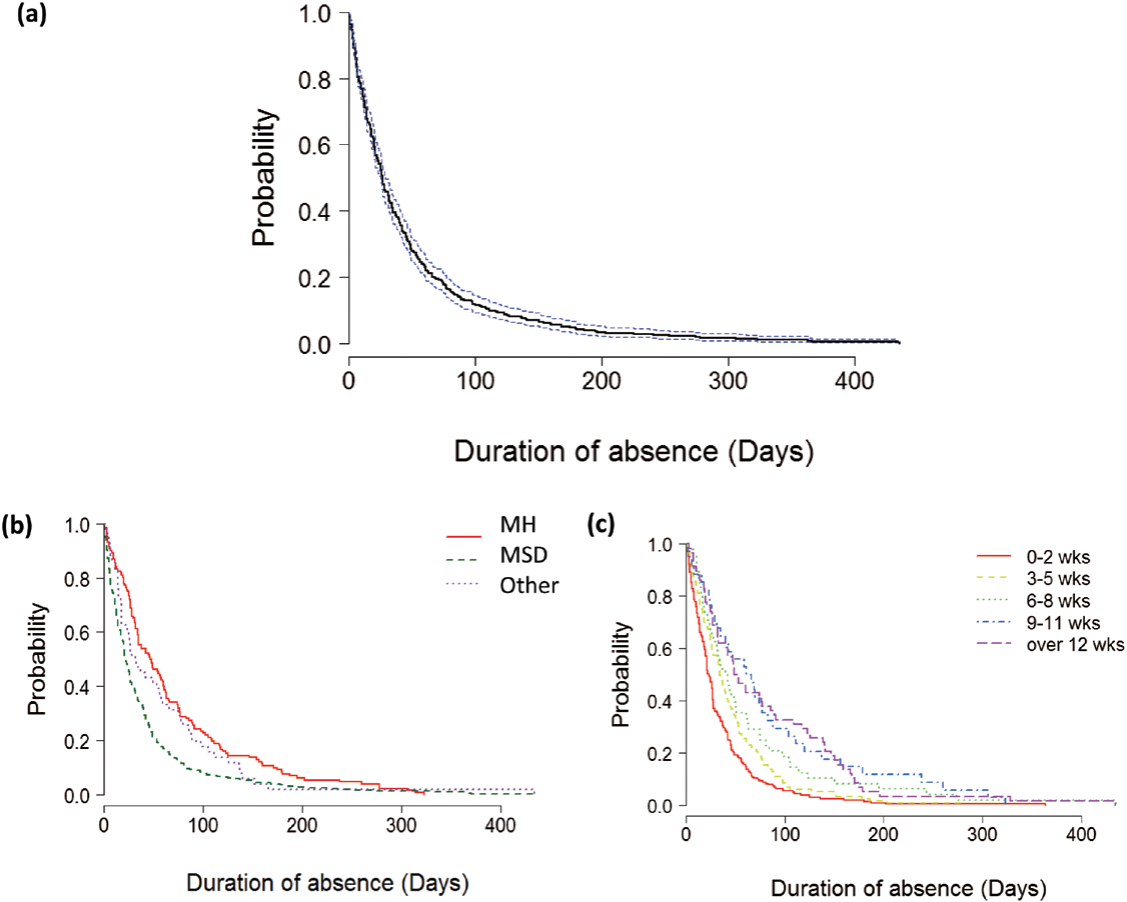
Survival analysis; (a) Kaplan–Meier RTW curve for all: number of days lost due to SA (solid line) with 95% CI (dotted line; *N* = 649); (b) Kaplan–Meier RTW curve by primary condition (MH = mental health cases; MSD = musculoskeletal cases); and (c) median length of time (days) being absent since entry assessment by the number of weeks absent before entry assessment.

Number of working days lost since entering WHSS was available for 649 cases. Overall, 50% were back to work in 27 days (95% CI: 25 to 29) (Figure 1a). Number of days lost was significantly higher in MH than MSD cases (*P* < 0.001); 50% of MH and MSD cases returned to work in 46 and 21 days, respectively. Number of days lost was also significantly higher for cases absent longer prior to entering WHSS (*P* < 0.001).

The best-fit model for SA duration (Table 2) included age, length of absence before entering WHSS, primary condition and discharge time. Gender, SIMD, occupation and general health status at entry were not significant. Age added almost 5 days to SA duration while in the programme for every 10-year age category. Moving up in SA duration prior to entry categories, 10 days were added to SA duration while in the programme. Duration of SA reduced by 10 days for cases presenting with MSD compared to MH conditions. Longer SA periods while in WHSS were associated with longer programme durations; for every 10 additional days in the programme, 2 days of SA were added.

**Table 2.** Model result of ARIMA model for duration of SA of referrals

Model parameters	Estimate (days)	Standard error of estimates	*Z*-statistic	*P*-value
Age (years)	0.49	0.16	3.04	<0.01
Duration of SA prior to entering the programme^a^ (ref = 0–2 weeks)	9.91	1.81	5.47	<0.001
Primary condition (ref = MH)	−10.60	3.65	2.90	<0.01
Discharge time (days)	0.22	0.03	8.23	<0.001

^a^SA duration prior to entry assessment was re-coded for the analysis (1 = 0 to 2 weeks; 2 = 3 to 5 weeks; 3 = 6 to 8 weeks; 4 = 9 to 11 weeks; 5 = over 12 weeks).

All health measures recorded a statistically significant beneficial change between entry and discharge (*P* < 0.001) (Table 3); the change in HADS scores for MH cases was greater than for MSD cases, while the changes in the other measures were more similar when comparing by health condition.

**Table 3.** Average changes in health measure scores

Measure	Pre-intervention mean score	Post-intervention mean score	Average change in score	Number	95% CI
EQ-5D index					
All completers	0.51	0.81	0.30	5590	[0.29 to 0.31]
MSD cases	0.50	0.81	0.31	4749	[0.29 to 0.31]
MH cases	0.58	0.84	0.26	646	[0.26 to 0.29]
EQ-5D VAS score					
All completers	59.1	80.0	22.5	5472	[22.49 to 22.50]
MSD cases	60.6	80.8	22.5	4653	[22.49 to 22.50]
MH cases	48.8	76.2	30.0	631	[27.50 to 30.00]
COPM^a^ Performance score					
All completers	3.84	7.54	3.70	3771	[3.99 to 4.00]
MSD cases	3.91	7.62	3.71	3182	[4.00 to 4.04]
MH cases	3.27	7.26	3.99	457	[4.00 to 4.50]
COPM^a^ Satisfaction score					
All completers	2.87	7.44	5.00	3754	[4.99 to 5.00]
MSD cases	2.91	7.53	5.00	3166	[5.00 to 5.17]
MH cases	2.46	7.18	5.00	457	[4.75 to 5.25]
HADS^b^ anxiety score					
All completers	7.36	4.04	−3.32	1696	[−4.00 to −3.50]
MSD cases	5.57	3.26	−2.31	1203	[−3.00 to −2.50]
MH cases	12.67	6.18	−6.50	400	[−7.50 to −6.50]
HADS^b^ depression score					
All completers	5.94	2.80	−3.14	1696	[−3.50 to −3.00]
MSD cases	4.68	2.33	−2.35	1203	[−3.00 to −2.50]
MH cases	9.65	3.98	−5.67	400	[−6.50 to −5.50]

^a^COPM Performance and Satisfaction scores range from 0 to 10; a higher score represents better performance, and better satisfaction.

^b^HADS scores range from 0 to 21, while 0–7 is considered ‘normal’, 8–10 ‘borderline’ and 11–21 is ‘caseness’. Note also that a negative change in score for the HADS anxiety and depression scores indicates an improvement.

In total, 88% (*n* = 4920) of cases improved their EQ-5D index score by an average of 0.35 (*P* < 0.001). By primary health condition, 89% of MSD and 84% of MH cases improved their EQ-5D score, with a slightly greater increase for MSD cases (Figure S2a, available as Supplementary data at *Occupational Medicine* Online). By absence status at entry (Figure S2b, available as Supplementary data at *Occupational Medicine* Online), 88% of those at work (*n* = 4267) and 88% of those absent (*n* = 1323) improved their EQ-5D score, by 0.28 and 0.35, respectively. Similarly, 81% improved their EQ-5D VAS scores by an average score of 27.8 points (*P* < 0.001). Of those who improved their score MH cases demonstrated a 6-point greater change compared with MSD cases (Figure S2c, available as Supplementary data at *Occupational Medicine* Online). Mean VAS score was higher both at entry and discharge for MSD compared with MH cases (Table 3). By absence status at entry (Figure S2d, available as Supplementary data at *Occupational Medicine* Online), a greater proportion of those who were absent at entry improved their VAS score, than those who were at work.

HADS scores show nearly one-third of cases (29%) had ‘caseness’ anxiety status at entry, changing to just 8% at discharge (Table S3a, available as Supplementary data at *Occupational Medicine* Online). In total, 574 cases (34%) transitioned to healthier anxiety categories, with 17% transitioning from the worst state ‘caseness’ to normal (Table S3a, available as Supplementary data at *Occupational Medicine* Online). By primary health condition, 23% of MSD (Table S3b, available as Supplementary data at *Occupational Medicine* Online) and 69% of MH cases (Table S3c, available as Supplementary data at *Occupational Medicine* Online) moved to a healthier anxiety state. Improvement in scores was greater for MH (89% improved by an average of 8%, *n* = 400) compared with MSD cases (67% improved by an average of 4.1, *n* = 1203). The change in scores was also greater for those absent at entry (80% improved by an average of 5.9, *n* = 490) compared with those at work (70% improved by an average of 4.7, *n* = 1206) (Table S3d and S3e, available as Supplementary data at *Occupational Medicine* Online).

A ‘caseness’ status for depression was reported by 18% at entry and dropped to 5% at discharge (Table S4a, available as Supplementary data at *Occupational Medicine* Online). A total of 455 (27%) cases transitioned to a healthier category, with 12% transitioning from the worst category ‘caseness’ to ‘normal’. Improvement in scores was greater for MH (85% improved by an average of 7%, *n* = 400) compared with MSD cases (66% improved by an average of 4.0, *n* = 1203) (Table S4b and S4c, available as Supplementary data at *Occupational Medicine* Online). The change in scores was also greater for those absent at entry (79% improved by an average of 6.1, *n* = 490) compared with those at work (69% improved by an average of 4.3, *n* = 1206) (Table S4d and S4e, available as Supplementary data at *Occupational Medicine* Online).

COPM Performance scores increased for 89% of cases (*n* = 3771) by 4.2 points on average. Improvements were similar for both MSD and MH cases (Figure S3a, available as Supplementary data at *Occupational Medicine* Online); and greater for those absent at entry than those at work at entry (Figure S3b, available as Supplementary data at *Occupational Medicine* Online).

Similarly, 90% of cases improved their COPM Satisfaction score (*n* = 3754) by 5.1 on average. Improvements were similar for both MSD and MH cases (Figure S3c, available as Supplementary data at *Occupational Medicine* Online); and greater for those absent at entry than those at work at entry (Figure S3d, available as Supplementary data at *Occupational Medicine* Online).

**Figure 2.**
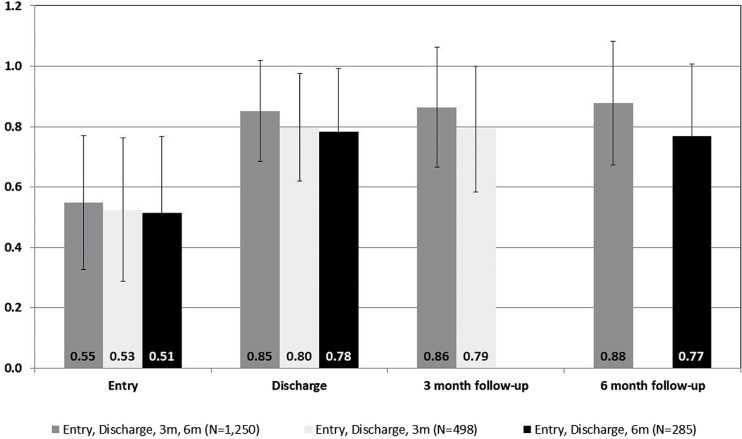
EQ-5D index score for entry, discharge, 3 and 6 months post-discharge.

Altogether 2033 cases provided EQ-5D follow-up data at ~3 months, 6 months, or both 3 and 6 months after discharge and these figures demonstrate a sustained improvement in EQ-5D scores at 3 and 6 months (Figure 2).

## Discussion

All health measures showed significant improvements from entry to discharge in health and functional ability; the majority of WHSS cases experienced health benefits. COPM scores significantly improved in 90% of cases, evidencing positive impacts on functional capacity and coping. The 3- and 6-month follow-ups provide evidence of the sustainability of the health improvements seen at discharge, and of cases remaining in work and working normal working hours after leaving WHSS.

An integral part of the case management process was to identify co-morbidities, which were present in 24% and 15% of cases with MH and MSD conditions, respectively. Identifying these co-morbidities will have helped case managers to provide more holistic care. This is evident in the HADS scores of individuals with MSD primary conditions where there was a 23% and 17% reduction of anxiety and depression symptoms, respectively. Co-morbidities are not always recognized in routine care but are likely to influence clinical and functional outcomes.

An important finding was the relationship between age and SA duration, with there being on average five more days of absence for every 10 years of age. Older workers may have longer SA, although generally have fewer SA episodes [20]. This finding indicates the need for improved OH and routine care for older workers.

The majority of cases in WHSS had an MSD (84%), which does not appear to reflect the fact that common MH disorders are a leading cause of SA [21]. This may suggest that a significant proportion of the workforce experiencing a MH condition may not be accessing this service. Ways of addressing this should be considered in any future programmes. Health improvements and RTW outcomes were generally better for MH than MSD cases, although they generally entered the programme with worse health scores and longer absence durations prior to entry.

The uniqueness and richness of this data set, covering an often hard-to-reach population—i.e. SME employees with health issues—are significant study strengths. Using secondary routine data, we analysed changes in health, well-being and work ability pre-/post-intervention. The study included a large sample, a large number of socio-demographic variables and the use of up to three standardized health/function assessment tools. Cases broadly represented the demographics of Scottish workers in terms of gender [22], while the service supported more older workers (>50 years) than the proportion reflected in Scottish employment statistics [22]. This is important given that older workers may have greater needs for such services in light of policies to extend working lives. The study covered the two leading causes of SA and work incapacity [6]. This highlights the need for services supporting those with MSD and MH conditions affecting work ability.

As is the case in many service evaluations, the lack of a control population is the main study limitation. Discharge data were only available for 60% of eligible cases, primarily due to not being able to contact the non-completers (60% of non-completers), or their voluntary withdrawal (15%). While the results demonstrate an improvement in health status that was sustained at 3 and 6 months post-discharge and an overall improvement in work status, the lack of a control means the effectiveness of WHSS cannot be assessed, as it is not possible to measure the health and employment outcomes for individuals who did not receive this service, and those who did not complete it. A new musculoskeletal service introduced at the end of 2011 in Scotland, re-routed MSD referrals to WHSS. These referrals increased the number of MSD cases in WHSS. There were no similar routes for identifying potential cases with MH conditions, which may partly account for the higher proportion of MSD cases.

In summary, this evaluation demonstrated a positive change in all health measures for WHSS participants at discharge, which was sustained at the 3- and 6-month follow-up. Of the available data, 93% of the total number of cases remained in work or returned to work by discharge. SA of SME employees can have detrimental impacts not only on individuals but also on employers. Evaluations of services such as WHSS can significantly impact services and policies; however, the evaluation process needs to be embedded in the programme development and implementation stages, to ensure that effectiveness and cost-effectiveness can be assessed. These results can inform the future delivery of national services with similar aims, and suggest the encouragement of early access to rehabilitation services.

**Key points**

- This study was a 4-year evaluation of the national Working Health Service Scotland that provides support to small and medium-sized enterprise employees with a health condition affecting their ability to work.
- The results demonstrate a positive change in all health measures (sustained at 3- and 6-month follow-up) following programme completion.
- Encouragement of early access to rehabilitation services to facilitate a quicker return-to-work for employees of small and medium-sized enterprises is suggested.

## Funding

E.D. was also supported by the MRC Strategic Award MC_PC_13027.

## Supplementary Material

kqx186_suppl_Supplementary_Tables
