# Supplementary material for: Working Health Services Scotland: a 4-year evaluation
Source: Occup Med (Lond). 2018 Jan 30;68(1):38–45. doi: 10.1093/occmed/kqx186 (PMC5927139; doi:10.1093/occmed/kqx186)
Supplement: kqx186_suppl_Supplementary_Tables [file kqx186_suppl_supplementary_tables.doc]

Table S1: Secondary health conditions of cases

| **Primary health condition** | **Secondary health condition** | | | |
| --- | --- | --- | --- | --- |
|  | MSD  n (%) | MH  n (%) | Other  n (%) | Total  n (%) |
| MSD (N=9,935) | 894 (9) | 397 (4) | 298 (3) | 1590 (16) |
| MH (N=1,434) | 86 (6) | 201 (14) | 57 (4) | 344 (24) |

Table S2: Duration in programme (entry assessment to discharge) (N=7,008)

| Duration in programme (days) | N (%) |
| --- | --- |
| 0 days | 21 (0.3) |
| 1 – 30 days | 266 (4) |
| 31 – 90 days | 2684 (38) |
| 91 – 180 days | 2873 (41) |
| 181 – 365 days | 1023 (15) |
| Over 365 days | 126 (2) |

*Table S3a: HADS anxiety status at pre- and post-intervention*

|  | | **Discharge** | | | Total  n (%) |
| --- | --- | --- | --- | --- | --- |
|  |  | Normal  n (%) | Borderline  n (%) | Caseness  n (%) |  |
| **Entry** | Normal n (%) | 926 (55%) | 29 (2) | 13 (1) | 968 (57) |
|  | Borderline n (%) | 182 (11) | 35 (2) | 12 (1) | 229 (14) |
|  | Caseness n (%) | 288 (17) | 104 (6) | 107 (6) | 499 (29) |
|  | Total n (%) | 1,396 (82) | 168 (10) | 132 (8) | 1,696 (100) |

*HADS scores range from 0 to 21, while 0-7 is considered normal, 8-10 borderline and 11-21 is ‘caseness’.

*Table S3b: HADS anxiety status at pre- and post-intervention for MSD cases*

|  | | **Discharge** | | | Total  n (%) |
| --- | --- | --- | --- | --- | --- |
|  |  | Normal  n (%) | Borderline  n (%) | Caseness  n (%) |  |
| **Entry** | Normal n (%) | 820 (68) | 23 (2) | 11 (1) | 854 (71) |
|  | Borderline n (%) | 125 (10) | 22 (2) | 9 (1) | 156 (13) |
|  | Caseness n (%) | 101 (8) | 48 (4) | 44 (4) | 193 (16) |
|  | Total n (%) | 1,046 (87%) | 93 (8%) | 64 (5) | 1,203 (100) |

*Table S3c: HADS anxiety status at pre- and post-intervention for MH cases*

|  | | **Discharge** | | | Total  n (%) | |
| --- | --- | --- | --- | --- | --- | --- |
|  |  | Normal  n (%) | Borderline  n (%) | Caseness  n (%) |  |  |
| **Entry** | Normal n (%) | 56 (14) | 3 (1) | 1 (0.3) | 60 (15) |  |
|  | Borderline n (%) | 47 (12) | 10 (3) | 2 (1) | 59 (15) |  |
|  | Caseness n (%) | 175 (44) | 52 (13) | 54 (14) | 281 (70) |  |
|  | Total n (%) | 278 (70) | 65 (16) | 57 (14) | 400 (100) |  |

*Table S3d: HADS anxiety status at pre- and post-intervention for those at work at entry*

|  | | **Discharge** | | | Total  n (%) |
| --- | --- | --- | --- | --- | --- |
|  |  | Normal  n (%) | Borderline  n (%) | Caseness  n (%) |  |
| **Entry** | Normal n (%) | 746 (62) | 23 (2) | 8 (1) | 777 (64) |
|  | Borderline n (%) | 123 (10) | 28 (2) | 2 (0) | 153 (13) |
|  | Caseness n (%) | 164 (14) | 61 (5) | 52 (4) | 276 (23) |
|  | Total n (%) | 1,032 (86) | 112 (9) | 62 (5) | 1,206 (100%) |

*Table S3e: HADS anxiety status at pre- and post-intervention for those absent at entry*

|  | | **Discharge** | | | Total  n (%) |
| --- | --- | --- | --- | --- | --- |
|  |  | Normal  n (%) | Borderline  n (%) | Caseness  n (%) |  |
| **Entry** | Normal n (%) | 180 (37) | 6 (1) | 5 (1) | 191 (39) |
|  | Borderline n (%) | 59 (12) | 7 (1) | 10 (2) | 76 (16) |
|  | Caseness n (%) | 125 (26) | 43 (9) | 55 (11) | 223 (46) |
|  | Total n (%) | 364 (74) | 56 (11) | 70 (14) | 490 (100) |

*Table S4a: HADS depression status at pre- and post-intervention*

|  | | **Discharge** | | | Total  n (%) |
| --- | --- | --- | --- | --- | --- |
|  |  | Normal  n (%) | Borderline  n (%) | Caseness  n (%) |  |
| **Entry** | Normal n (%) | 1,100 (65) | 28 (2) | 13 (1) | 1,141 (67) |
|  | Borderline n (%) | 211 (12) | 27 (2) | 11 (1) | 249 (15) |
|  | Caseness n (%) | 209 (12) | 35 (2) | 62 (4) | 306 (18) |
|  | Total n (%) | 1,520 (90) | 90 (5) | 86 (5) | 1,696 (100) |

*Table S4b: HADS depression status at pre- and post-intervention for MSD cases*

|  | | **Discharge** | | | Total  n (%) |
| --- | --- | --- | --- | --- | --- |
|  |  | Normal  n (%) | Borderline  n (%) | Caseness  n (%) |  |
| **Entry** | Normal n (%) | 916 (76) | 19 (2) | 6 (1) | 941 (78) |
|  | Borderline n (%) | 113 (9) | 17 (1) | 6 (1) | 136 (11) |
|  | Caseness n (%) | 82 (7) | 14 (1) | 30 (3) | 126 (11) |
|  | Total n (%) | 1,111 (92) | 50 (4) | 42 (4) | 1,203 (100) |

*Table S4c: HADS depression status at pre- and post-intervention for MH cases*

|  | | **Discharge** | | | Total  n (%) |
| --- | --- | --- | --- | --- | --- |
|  |  | Normal  n (%) | Borderline  n (%) | Caseness  n (%) |  |
| **Entry** | Normal n (%) | 127 (32) | 7 (2) | 5 (1) | 139 (35) |
|  | Borderline n (%) | 85 (21) | 8 (2) | 3 (1) | 96 (24) |
|  | Caseness n (%) | 119 (30) | 17 (4) | 29 (7) | 165 (41) |
|  | Total n (%) | 331 (83) | 32 (8) | 37 (9) | 400 (100) |

*Table S4d: HADS depression status at pre- and post-intervention for those who were at work at entry*

|  | | **Discharge** | | | Total  n (%) |
| --- | --- | --- | --- | --- | --- |
|  |  | Normal  n (%) | Borderline  n (%) | Caseness  n (%) |  |
| **Entry** | Normal n (%) | 892 (74) | 19 (2) | 6 (1) | 917 (76) |
|  | Borderline n (%) | 124 (10) | 18 (2) | 6 (1) | 148 (12) |
|  | Caseness n (%) | 105 (9) | 14 (1) | 22 (2) | 141 (12) |
|  | Total n (%) | 1,121 (93) | 51 (4) | 34 (3) | 1,206 (100) |

*Table S4e: HADS depression status at pre- and post-intervention for those who were absent at entry*

|  | | **Discharge** | | | Total  n (%) |
| --- | --- | --- | --- | --- | --- |
|  |  | Normal  n (%) | Borderline  n (%) | Caseness  n (%) |  |
| **Entry** | Normal n (%) | 208 (42) | 9 (2) | 7 (1) | 224 (46) |
|  | Borderline n (%) | 87 (18) | 9 (2) | 5 (1) | 101 (21) |
|  | Caseness n (%) | 104 (21) | 21 (4) | 40 (8) | 165 (34) |
|  | Total n (%) | 399 (81) | 39 (8) | 52 (11) | 490 (100) |


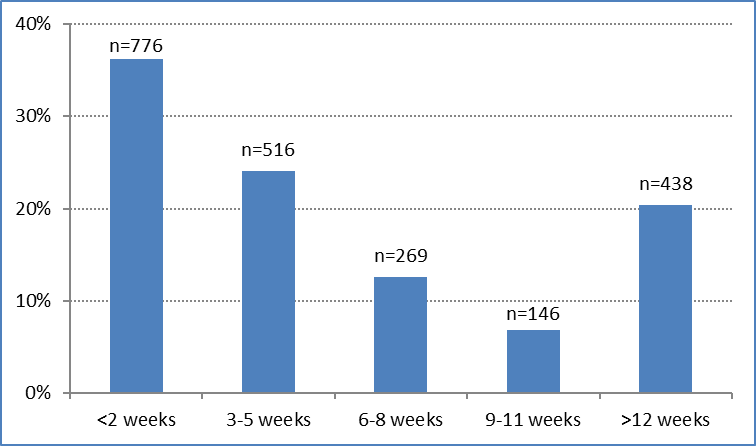


Figure S1: Percentage and number (n) of WHSS cases by the number of weeks on sick leave before their entry assessment (N=2,145)

| 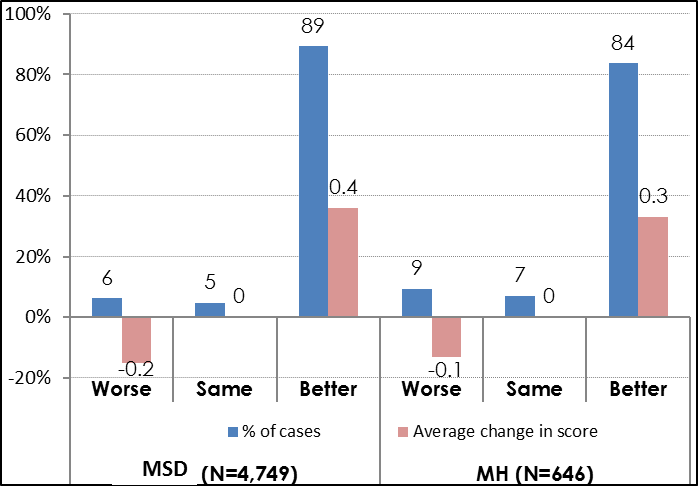    FigureS2a: Change in EQ-5D index values shown for MSD and MH cases |
| --- |
| 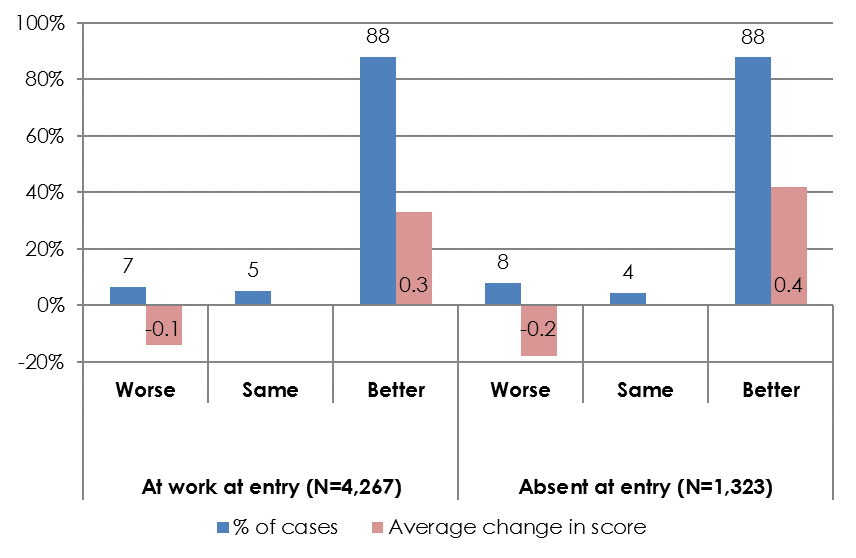  Figure S2b: Change in EQ-5D index values shown for those at work / absent at entry |
| 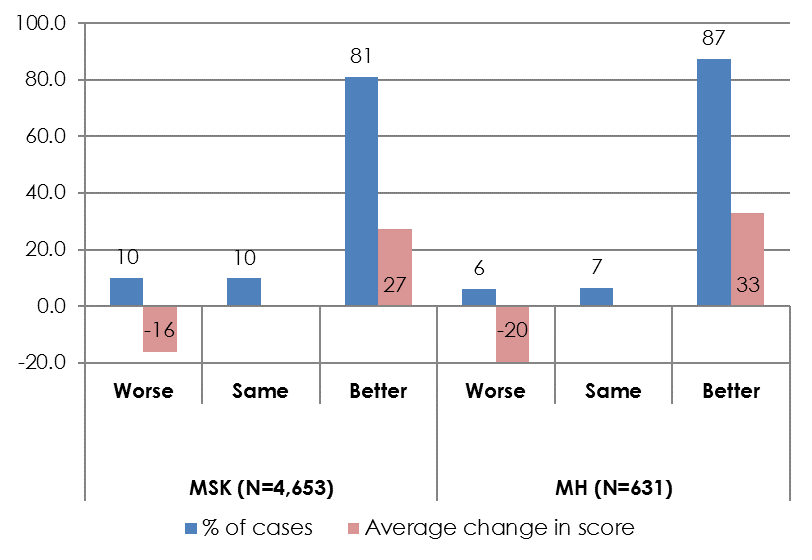  **MSD**  Figure S2c: Change in EQ-5D VAS scores shown for MSD and MH cases |
| 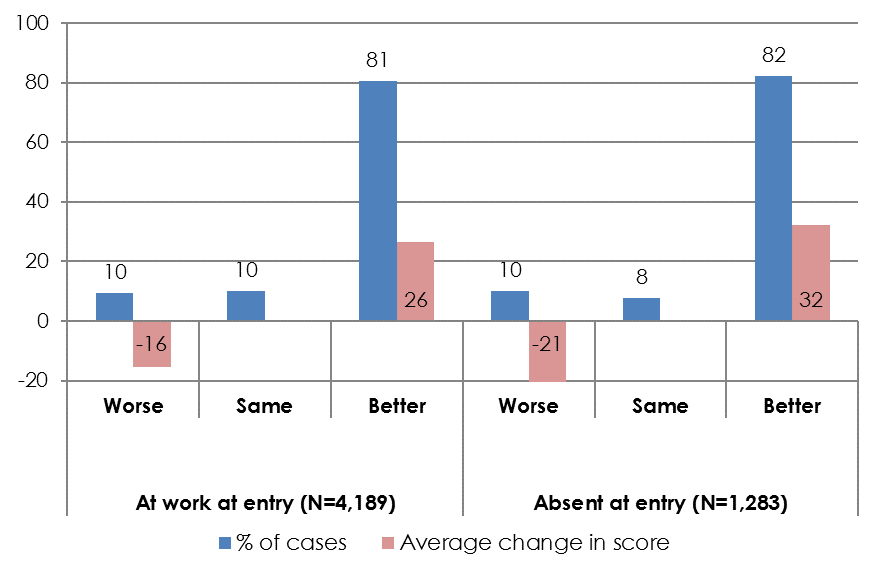  Figure S2d: Change in EQ-5D VAS scores shown for those at work / absent at entry |


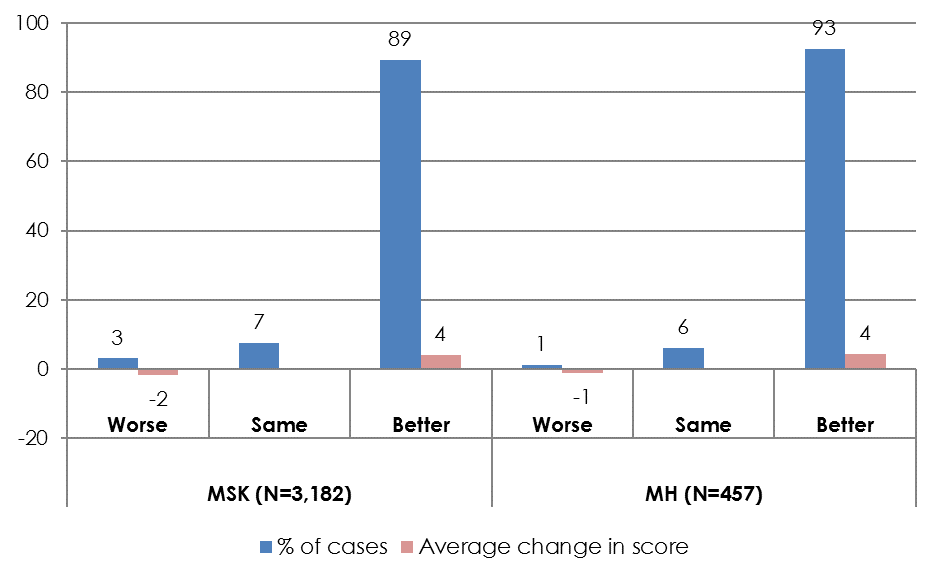


**MSD**

Figure S3a: Change in COPM Performance scores shown for MSD and MH cases


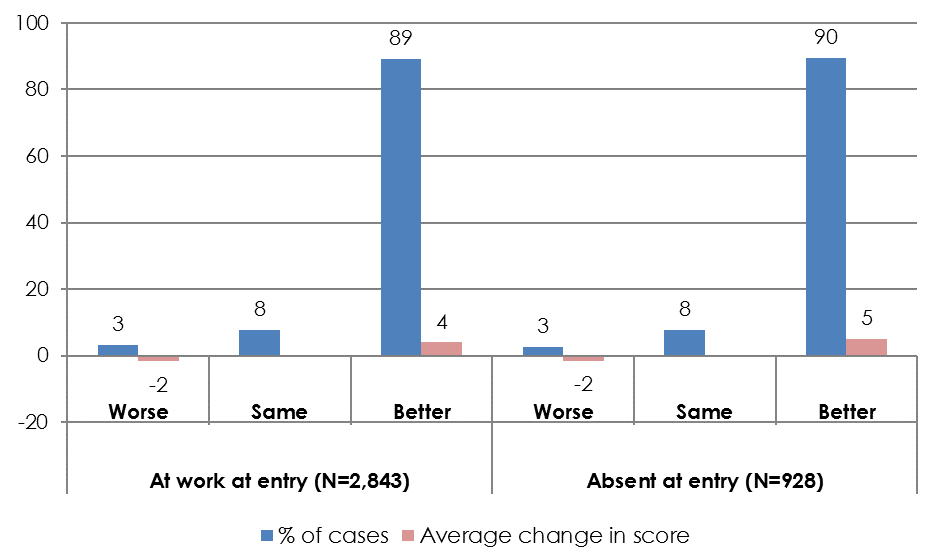


Figure S3b: Change in COPM Performance scores shown for those at work / absent at entry


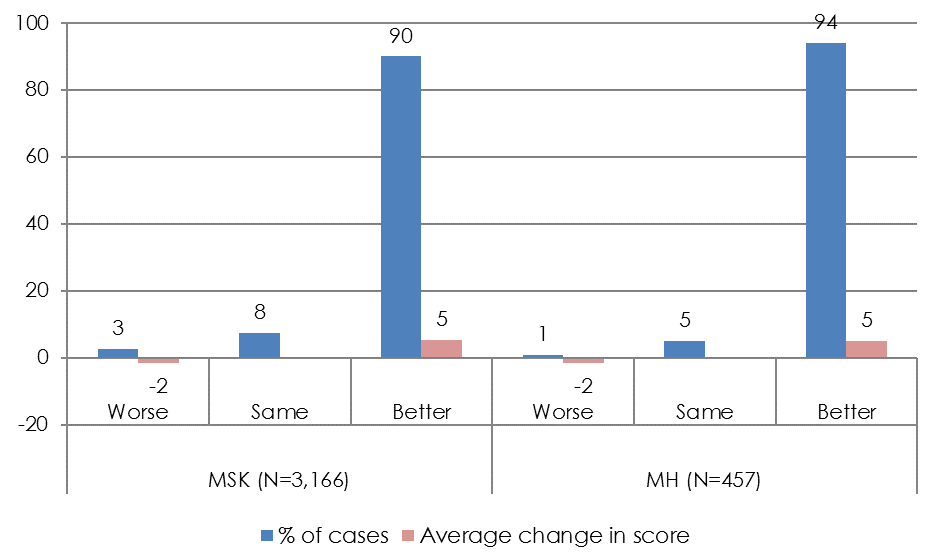


MSD

Figure S3c: Change in COPM Satisfaction scores shown for MSD and MH cases


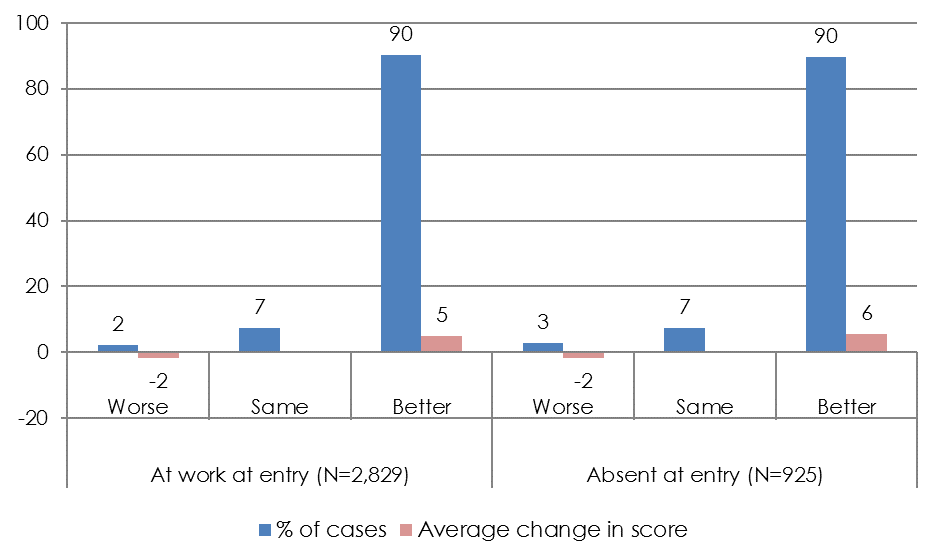


**Figure S3d: Change in COPM Satisfaction scores shown for those at work / absent at entry**
